# Similarities and Differences in the Outsiders and Insiders' Visual Preferences on Sacred Landscape

**DOI:** 10.3389/fpsyg.2022.743933

**Published:** 2022-07-07

**Authors:** Chang Li, Shanwei Ge, Ruiying Wang

**Affiliations:** School of Architecture and Urban Planning, Suzhou University of Science and Technology, Suzhou, China

**Keywords:** landscape assessment, religious settings, eye tracking, visual landscape, insider study, outsider study

## Abstract

Previous studies have reported religious and non-religious people as having different psychological experiences when visiting sacred landscapes; however, the visual consensus and differences between diverse groups visiting them have rarely been considered. This study used subjective preference evaluation and experimental eye tracking to assess the visual preferences of different groups regarding sacred landscapes. Overall, 48 photos of the Han Chinese Buddhist temples were selected as stimulus materials, including the categories of squares, architecture, waterscapes, and plants. In all, 90 participants were classified into two groups of outsiders and insiders to view the photos. The consensus and differences in their visual preferences and eye movement metrics were evaluated. The results showed that the two groups were more inclined toward the visual preference of religious architectures than the natural landscape that people usually prefer. Another noteworthy discovery revealed the significant differences between the outsiders and the insiders in viewing and evaluating sacred landscapes; the immersion effect explains this result. Specifically, the group with a higher interaction with the environment had greater visual experiences, easier visual information coding, and larger visual exploration range. In addition, this study revealed familiarity with the religious background facilitated achieving a higher consistency between the landscape preference scores and the eye movement metrics. These findings expand the theory of religious environment perception and provided important insights for subsequent research on sacred landscape planning and management.

## Introduction

Urban green spaces have several positive impacts on the physical and mental health of urban residents. Inspired by the Attention Restoration and Psycho-evolutionary theories (Ulrich et al., 1991; Kaplan, 1995), a growing number of studies have evaluated the effects of different urban green space types, including rivers, forests, parks, squares, and street greening on visual attraction, restoration potential, and stress recovery (Nordh et al., 2013; Amati et al., 2018; Cottet et al., 2018; Franěk et al., 2018). However, they have not sufficiently emphasized the specifics of particular environments. The sacred landscape, which is closely linked to nature and culture, is one of the aspects that needs to be explored further. Although this idea often brings serious discussion in anthropological studies, to the best of our knowledge, limited experimental studies have focused on the visual preference of different users regarding sacred landscapes as urban green spaces.

Although psychologists regard religion as an internal cognitive attitude and belief, the external physical environment plays an important role in religious life (Meagher, 2018). During the formation of many cities, sacred landscapes (e.g., churches, temples, and mosques) were physically embedded in faith-based communities, and the buildings that dominated the city center were usually religious monuments (Ysseldyk et al., 2016). Many researchers have recognized the differences between religious and secular spaces; they focused on the comparison between the physical environment and the environmental experience. Regarding the former, a sacred landscape was recognized as a place of biodiversity (Schaaf and Lee, 2006; Verschuuren et al., 2010), having unique architectural structure, garden style (Herzog et al., 2013; Nordh et al., 2017; Meagher, 2018), and rich soundscape (Tenngart Ivarsson and Hagerhall, 2008; Herzog et al., 2013; Zhang et al., 2016). In terms of the latter, researchers suggested that the physical structure of sacred landscapes (e.g., scale, magnitude, shape) was capable of eliciting transcendent emotions associated with spirituality (Joye et al., 2013; Meagher, 2018); moreover, the religious soundscape (e.g., sounds of nature, sounds of musical instruments, the singing of hymns) preferences were related to clarity (Zhang et al., 2016).

Several renowned studies such as the Environmental Preference, the Attention Restoration, and the Stress Recovery theories associated the physical environment of sacred landscapes to people's preferences; this indicated that sacred urban sites (e.g., churches, monasteries, temples, and cemeteries) were more popular among the public than the general artificial or natural ones (Ulrich, 1979; Kaplan and Kaplan, 1989; Ouellette et al., 2005; Ysseldyk et al., 2016). These theories explained the non-religious cognitive factors of people's preference for sacred landscapes, such as mystery, complexity, remoteness, charm, and acoustic comfort from the perspective of an overall wider population (general visitors).

Different from the ordinary urban green spaces, the main people who visited the sacred landscapes were believers (Terzidou et al., 2018). In recent years, extensive research has been conducted on the benefits of visiting religious spaces. Immersion in a religious environment was found to increase the well-being of religious group members (Sternberg, 2009), improve social self-esteem (Ysseldyk et al., 2016), enhance self-perceptions of both psychological and physical health (Ouellette et al., 2005; Eriksson and Wiklund-Gustin, 2014), and improve place identity and attachment (Mazumdar and Mazumdar, 2004); however, these benefits might be placebo effects (Ysseldyk et al., 2016).

Plenty of previous studies reported different user groups as having diverse background variables, such as environmental behaviors and attitudes, living environment, age range, education level, and professional knowledge that affected their way of evaluating visual landscapes (Sevenant and Antrop, 2010; Dupont et al., 2015; Pihel et al., 2015; Ode Sang et al., 2016). Although the research on tourists' and believers' visits to the sacred spaces of a city is relatively extensive, limited studies have attempted to link these two groups, especially regarding the comparison of their urban religious experiences. For example, the self-perceived discrepancies between Christians and atheists who visited churches (Ysseldyk et al., 2016), the different impressions of congregants and external observers toward worship settings (Meagher, 2018), and the diverse psychological perceptions of religious symbols in public places between religious and non-religious people (Bilewicz and Klebaniuk, 2013). In addition, in terms of research methodology, these studies mainly focused on the subjective evaluation of sacred landscape pictures and on-site environments. However, thus far, an objective measurement of the visual perceptual differences among different groups who observed sacred landscapes has been scarcely performed.

As a relatively new technology, eye tracking offers an objective method to examine different groups' observation of landscapes. It allows the recording of quantitative metrics of various eye movements while watching an image (e.g., fixation duration, number of fixations, number of saccades, saccade amplitude, scan-path length, blink rates, etc.), and evaluates discrepancies in the manner of viewing of experts and laymen (Dupont et al., 2014).

Recently, many studies have confirmed the effectiveness of eye tracking and offered valid results, such as the differences between expert and novice groups when assessing the biodiversity of harvested forests (Pihel et al., 2015), the visual perception discrepancies between experts and laymen when viewing landscape photos (Dupont et al., 2015), the disparity in visual recognition memory between expert image analysts and untrained viewers when observing aerial photographs (Šikl et al., 2019), and the visual differences between the potential Chinese and English rural tourists' landscape preferences (Ren, 2019).

Visual images have been proven to provide a medium for landscape evaluation in a comprehensible way; additionally, many studies have used them for evaluating sacred landscapes (Herzog et al., 2013; Ysseldyk et al., 2016; Meagher, 2018). However, existing research on sacred landscapes focuses on the restorative preferences and benefits of ordinary visitors and believers, rather than comparing the visual consensus and the group differences between them regarding perception of sacred landscapes. If the visual preferences of outsiders (tourists who visit sacred landscapes occasionally) and insiders (local residents who have an environmental attachment with sacred landscapes) viewing sacred landscapes are unknown, then making effective suggestions for the resource management of sacred landscape paradigms would be challenging.

Therefore, this study aimed to contribute new insights into the visual preferences of sacred landscape as urban green spaces, as well as to explore the perception of sacred landscapes combined with subjective assessment and eye tracking. The study purported to investigate the visual preference consensus and compare the differences between outsiders and insiders when viewing images of sacred landscape.

## Manuscript Formatting

### Stimulus Materials

The stimuli photos employed in this experiment were of the Han Chinese Buddhist temples, the most popular sacred landscape in Chinese cities. These temples usually have a vast history and visually appealing environment. Before the emergence of modern urban parks, they were the main urban green spaces for citizens to relax, regardless of whether or not they were believers. We surveyed 84 visitors from three Buddhist temples in Suzhou, Southeast China, and selected four types of landscapes that they were most concerned with, including waterscapes (*N* = 50), plants (*N* = 36), squares (*N* = 31), and architectural spaces (*N* = 22). Subsequently, 513 photos were taken in 12 urban Buddhist temples. In order to reduce the influence of factors such as the season, weather, and equipment, all images were recorded on a cloudy day during the same season (September–October 2020) with the same camera (Cannon EOS-M3).

**Figure 1:**
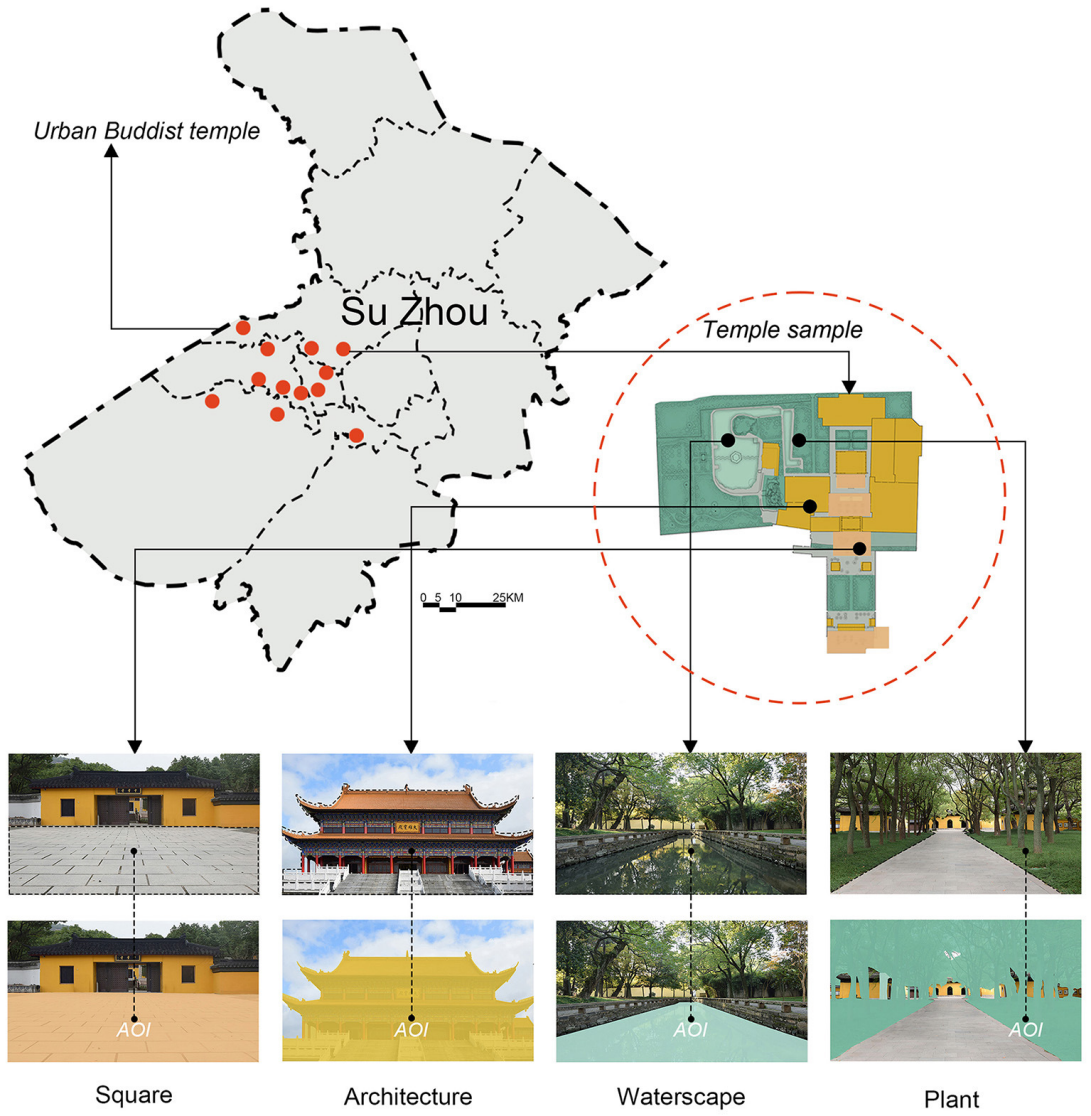
Locations of the sacred landscape sampling sites in Suzhou, and the four representative landscape types.

A series of photos were selected as experimental stimuli based on the opinions of three experts. First, all experts excluded the pictures that did not meet the typical landscape characteristics of the Han Chinese Buddhist temples on their computers. Second, images that were considered likely to interfere with the eye movement experiment, such as those with strange plants, trash cans, and human beings were also excluded. Finally, 48 photos were chosen by the experts. They were divided into four landscape types according to the characteristics presented: squares, architecture, waterscapes, and plants (Figure 1).

**Figure 2:**
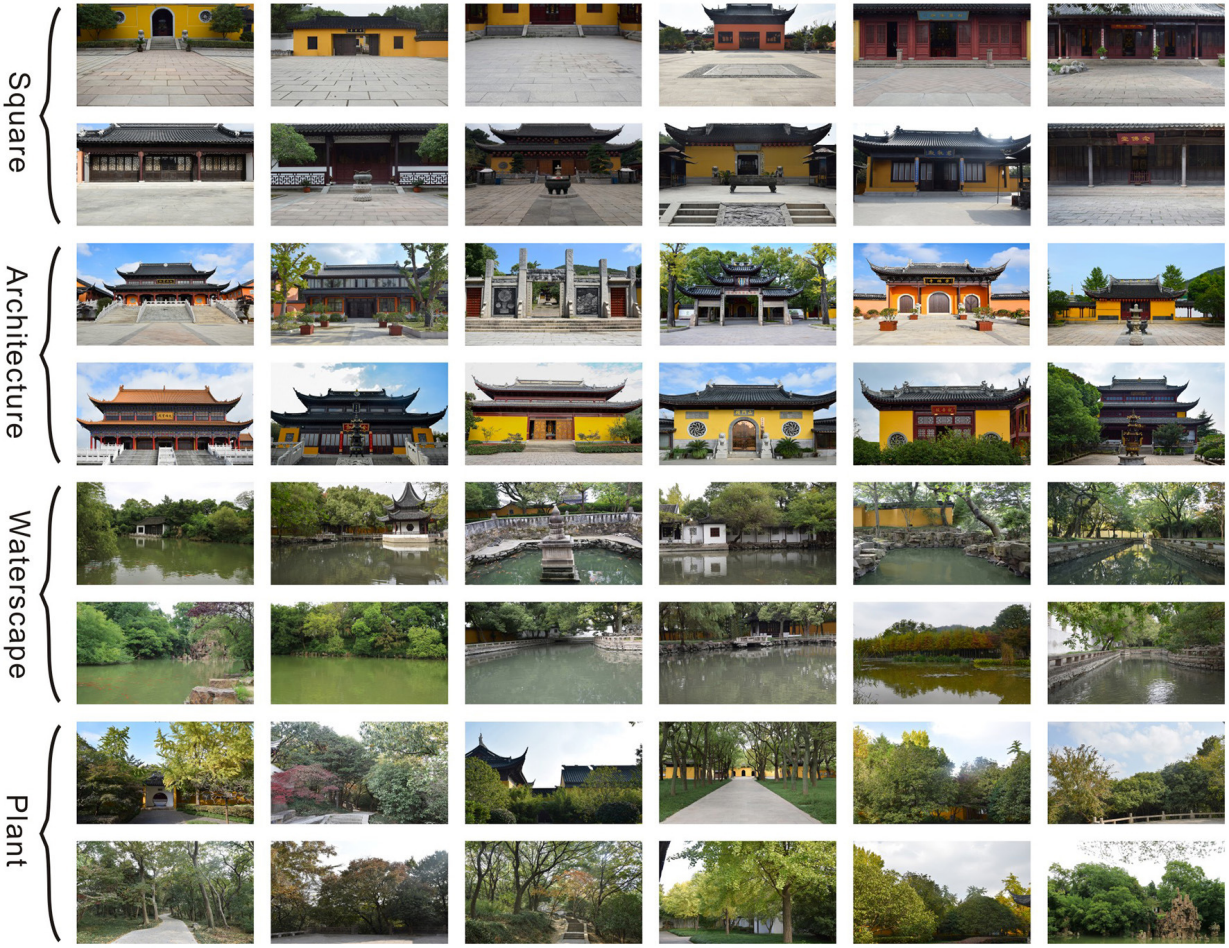
The stimuli photos.

In all, 12 photos were representative of the typical Buddhist buildings in the Chinese cities that had adopted historical styles, including the Buddha Hall, the Buddhist Foyer, and memorial arches. Further, 12 images depicted hard-paved squares that were usually located in front of the Buddha Hall for periodical Buddhist ceremonies. Next, 12 pictures comprised ponds representing typical water bodies in the Buddhist temples. The Han Chinese Buddhist doctrine forbids killing animals to eat their meat; thus, many Buddhist temples have Free Life Ponds for believers to release aquatic animals (e.g., fishes, crabs, and turtles) to let them live peacefully. The last 12 photos presented various types of plants that are also typical representatives of the Buddhist landscapes, such as *Ficus religiosa*, because Gautama (Buddha, the founder of Buddhism) is believed to have received enlightened under a *Ficus benghalensis* (Nene, 2000). All images were converted to a resolution of 1,920 × 1,080 pixels using the Adobe Photoshop CS 6 software; the brightness levels and the contrast balances were adjusted automatically (Figure 2).

### Participants

Two groups of respondents participated in the eye tracking experiment. Five respondents with poor eye tracking data (e.g., data loss caused by blinking) were excluded. Overall, 90 respondents completed the experiment, including 45 outsiders (14 male and 31 female) and insiders (16 male and 29 female) each. Insiders are local residents who have an environmental attachment with sacred landscapes. They are middle-aged residents of urbanized communities with common Buddhist identification, and they visit the Han Chinese Buddhist temples periodically (Merton, 1972). Referring to a previous study on the difference in landscape perception preferences of tourists and local residents (van den Berg et al., 1998; Scott, 2002), the inclusion criteria for the outsiders were that they were not Buddhists and had not visited a sacred landscape. They were recruited through the university's social platform, and had a mean age of 21.69 years (SD = 1.92). The insiders were mainly urban community residents and Buddhists. The mean monthly frequency of visiting the Han Chinese Buddhist temples near the community was 4.04 (SD = 0.51), and their mean age was 54.95 years (SD = 12.78). They were mainly recruited using the snowball method. Previous studies demonstrated that familiarity with images might affect gaze behavior (Franěk et al., 2018); thus, we excluded those participants who were familiar with Buddhist temples in the photos. All participants had a normal or corrected vision, and none of them reported visual impairments or other problems. The study sample was estimated through a priori power calculation with G-power version 3.1.9.2. The results of the calculation highlighted a study size of 43 each group [effect size *d* = 0.5, α err prob = 0.05, and power(1 – βerr prob) = 0.8].

### Apparatus

The study used the aSee pro reflective eye tracker (7Invensun Technology Ltd.) to measure eye movements with a sampling rate of 256 Hz. The experiment pictures were displayed using a computer (DELL OPTIPLEX 760) with a screen resolution of 1,920 × 1,080 pixels, and the screen diagonal was 58.42 cm. The eye tracker was arranged under the computer monitor. The presentation and data processing of the stimulation device was controlled by the aSee pro 3.2 software.

### Measurement

The eye movement metrics employed in this study were the time to first fixation, the average fixation duration, and the number of saccades. These metrics provided information about the main observation patterns in previous studies, such as attention to stimuli, recovery potential, attention, and so on. Among them, the time to first fixation was used to reflect the characteristics of the early stage of vision. The shorter the participants' time to first fixation in the area of interest (AOI), the higher the attention attracted (Hollingworth, 2009; Guo et al., 2016). The average fixation duration indicated the general attractiveness of the fixation points in the AOI. The harder it was for the participants to perceive the picture, the longer was their average time of staring at the fixation point (Franěk et al., 2018; Stevenson et al., 2019). The number of saccades was related to visual exploration; higher saccades were indicative of greater extensive inspection (Dupont et al., 2015).

The eye tracking experiment could measure the changes in the interviewees' eye movements when viewing a picture; however, it could not identify their subjective perceptions and emotions. Therefore, before the experiment, we collected the background information, such as age, gender, and religious views, of each respondent through questionnaires. After the experiment, they were requested to assess the landscape preferences of each picture using a 7-point Likert scale (ranging from −3 = Not at all to 3 = very much) as well as provide a specific explanation for it.

In summary, the combination of eye tracking and subjective evaluation provided an integrated method. The eye tracking data allowed us to gain insight into the landscape characteristics of sacred landscape that attracted the visual attention of outsiders and insiders. Furthermore, the surveys facilitated determining how the participants evaluated their landscape preferences.

### Procedure

All participants were tested separately. After the researcher introduced the experimental procedures, they signed the experimental consent form and provided brief demographic information. They were requested to sit 60 cm from the display. Before each experiment, a nine-point calibration procedure was used for the participant's eye tracking. Subsequently, 48 pictures were displayed in a random order, and each picture was presented for 10 s. Before the image appeared, the participants had to concentrate on the cross at the screen's center for 2 s. After the eye tracking experiment was completed, they were instructed to score and explain their preferences for each stimulus picture on another computer. The entire experiment lasted for about 30 min. Moreover, the participants were informed that they had the right to withdraw from the experiment at any time. All research procedures were approved by the university ethics committee.

### Data Analysis

According to the main landscape element types (squares, architectures, waterscapes, and plants) of the sacred landscapes, the AOI was specified for all stimulating pictures. The eye movement metrics and the landscape preference scores were statistically analyzed using SPSS 24.0. In order to investigate whether outsiders and insiders had different views on sacred landscapes, the mean values of the time to first fixation, the average fixation duration, the number of saccades, and the landscape preference scores of the two groups were used for a paired *t*-test comparison. If the eye movement metrics were not normally distributed, the non-parametric Mann–Whitney *U* test was performed. This test was based on ranks comparison and was employed to detect whether the observation ranks in one group (insiders) was evidently more or less than those in the other group (outsiders). The Cohen's *d* (Cohen, 1988) and η^2^ = *z*^2^/*N* (Coolican, 2009) were adopted to calculate the effect size (Small: >0.2 and <0.5; Medium: >0.5 and <0.8; Large: >0.8). The internal reliability of the landscape preference scores was analyzed using Cronbach's alpha. Furthermore, the Pearson correlation coefficient measurement was employed to assess the correlation difference between the landscape preference scores and the corresponding eye movement metrics. Finally, the scoring reasons for each stimulus picture were transcribed, coded, classified, and frequency counted. The statistical analysis was completed using SPSS 24.0.

## Results

### Landscape Preferences

**Figure 3:**
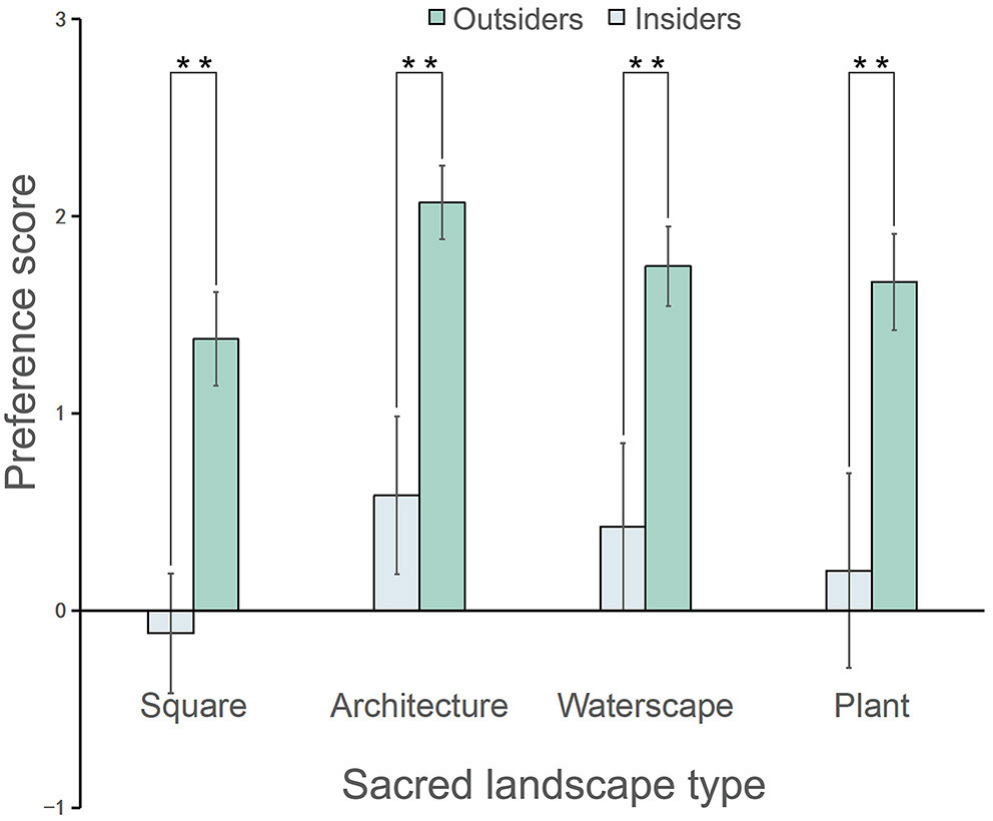
Comparison of the sacred landscape preference scores of the outsiders and insiders (*p < 0.05, **p < 0.01); the data are shown as means (SD). *N* = 45 for each group.

As shown in Table 1, Figure 3, three sacred landscapes were found to have a positive effect on the landscape preference scores; however, the square was evaluated negatively and positively by the outsiders and the insiders, respectively. The preference scores of the four types of sacred landscapes in the descending order were architectures, waterscapes, plants, and squares, as assessed by both the groups. However, the paired *t*-test analysis reported a significant difference in the sacred landscape preference scores across the groups (*t*(47) = 32.35, *p* < 0.001, Cohen's *d* = 3.52), where those of the outsiders (0.27 ± 0.48) were even lesser than the insiders (1.72 ± 0.33). More specifically, in Figure 3, the sacred landscape preference scores of the former were significantly lower for squares (outsiders: −0.12 ± 0.30; insiders: 1.38 ± 0.24, *p* < 0.001, Cohen's *d* = 5.52), architectures (outsiders: 0.58 ± 0.40; insiders: 2.07 ± 0.19, *p* < 0.001, Cohen's *d* = 4.76), waterscapes (outsiders: 0.43 ± 0.42; insiders: 1.75 ± 0.20, *p* < 0.001, Cohen *d*'s *d* = 4.01) and plants (outsiders: 0.20 ± 0.49; insiders: 1.67 ± 0.24, *p* < 0.001, Cohen's *d* = 3.81).

**Table 1:** Results for outsiders and insiders: the mean landscape preference scores with associated paired *t*-test analysis; and mean values of the eye movement metrics, with their non-parametric Mann–Whitney *U* tests.

	**Outsiders**		**Insiders**		***p***	**Effect size η^2^**
	**M**	**SD**	**M**	**SD**		
Preference score	0.27	0.48	1.72	0.33	<0.001	0.75
Time to first fixation (ms)	189.63	65.03	141.73	52.64	<0.001	0.45
Mean fixation duration (ms)	191.87	67.87	167.97	55.65	<0.001	0.45
Number of saccades (*n*)	11.94	10.01	12.03	8.93	0.436	0.01

*N = 45 for each group*.

### Eye-Tracking Measures

The non-parametric Mann–Whitney *U* test indicated a significant difference between the two groups with respect to the eye movement metrics (Table 1), including the time to first fixation, the mean fixation duration, and the number of saccades.

#### Time to First Fixation

**Figure 4:**
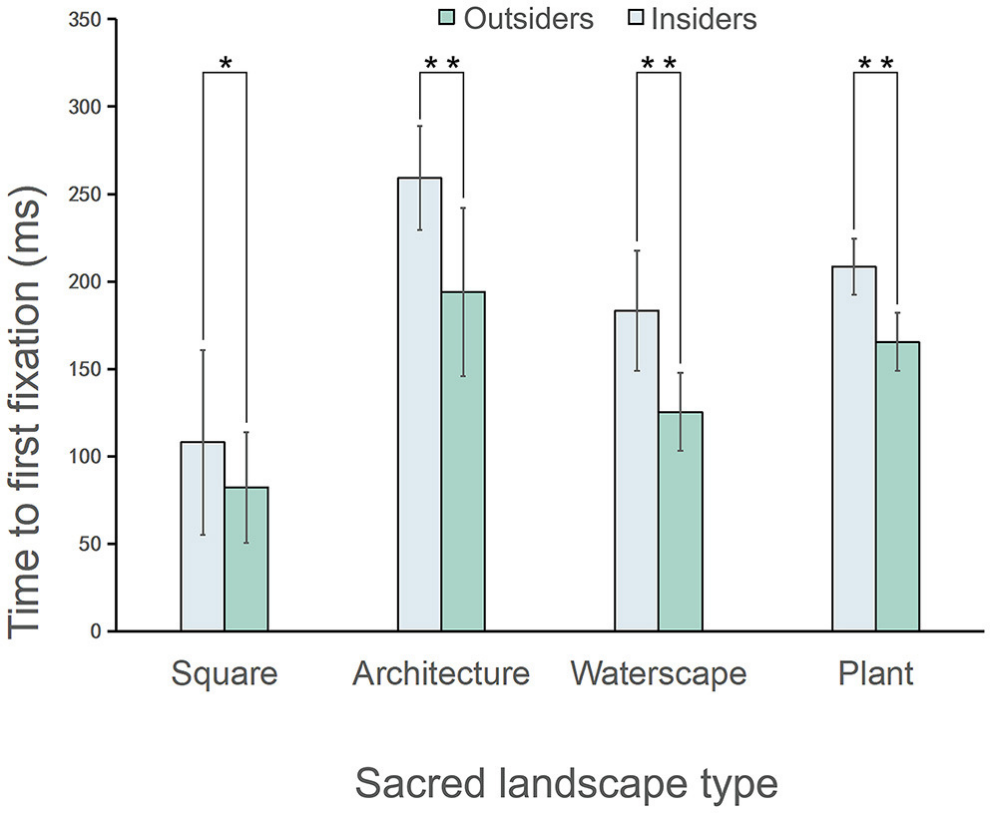
Comparison of the time to first fixation between the outsiders and insiders (*p < 0.05, **p < 0.01); the data are shown as means (SD). *N* = 45 for each group.

The difference in the time to first fixation (for all images) between outsiders (189.63 ± 65.03) and insiders (141.73 ± 52.64) was significant (*z* = 4.65, *p* < 0.001, η^2^ = 0.45), with the former demonstrating higher values than the latter (Table 1). More specifically, as displayed in Figure 4, the time to first fixation value of the outsiders was significantly greater in the AOI of the squares (outsiders: 107.94 ± 52.80; insiders: 82.16 ± 31.54, *z* = 2.60, *p* < 0.05, η^2^ = 0.59), architectures (outsiders: 259.01 ± 29.80; insiders: 193.95 ± 48.23, *z* = 1.65, *p* < 0.01, η^2^ = 0.23), waterscapes (outsiders: 183.19 ± 34.27; insiders: 125.40 ± 22.23, *z* = 3.06, *p* < 0.001, η^2^ = 0.78), and plants (outsiders: 208.39 ± 16.15; insiders: 165.44 ± 16.65, *z* = 1.96, *p* < 0.01, η^2^ = 0.32).

#### Average Fixation Duration

**Figure 5:**
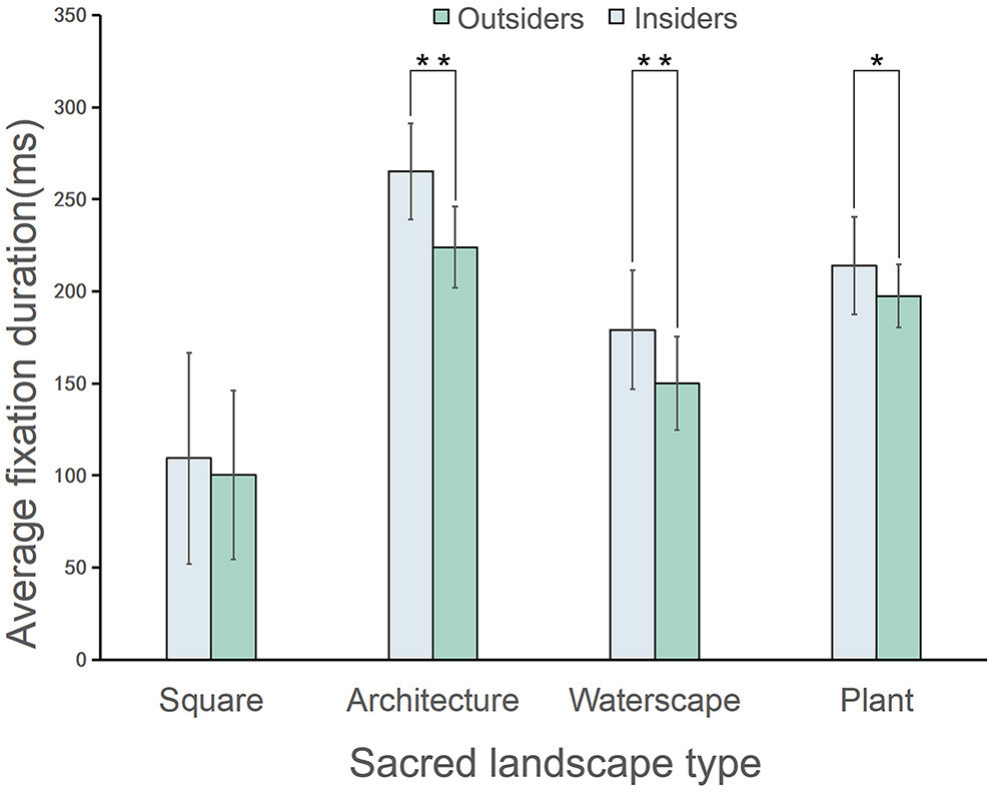
Comparison of the average fixation duration between the outsiders and the insiders (*p < 0.05, **p < 0.01); the data are shown as means (SD). *N* = 45 for each group.

The difference (*z* = 4.65, *p* < 0.001, η^2^ = 0.45) in the average fixation duration (for all images) of the outsiders (191.87 ± 67.87) and the insiders (167.97 ± 55.65) was significant, with the former indicating greater values than the latter (Table 1). As presented in Figure 5, regarding the specific AOI of each landscape type, the average fixation duration of the outsiders was significantly higher than that of the insiders for architectures (outsiders: 265.12 ± 26.08; insiders: 223.96 ± 22.18, *z* = 2.90, *p* < 0.01, η^2^ = 0.70), waterscapes (outsiders: 179.14 ± 32.22; insiders: 150.02 ± 25.28, *z* = 2.98, *p* < 0.01, η^2^ = 0.74), and plants (outsiders: 213.89 ± 26.41; insiders: 197.51 ± 17.14, *z* = 2.197, *p* < 0.01, η^2^ = 0.40), however, not for squares.

### Number of Saccades

**Figure 6:**
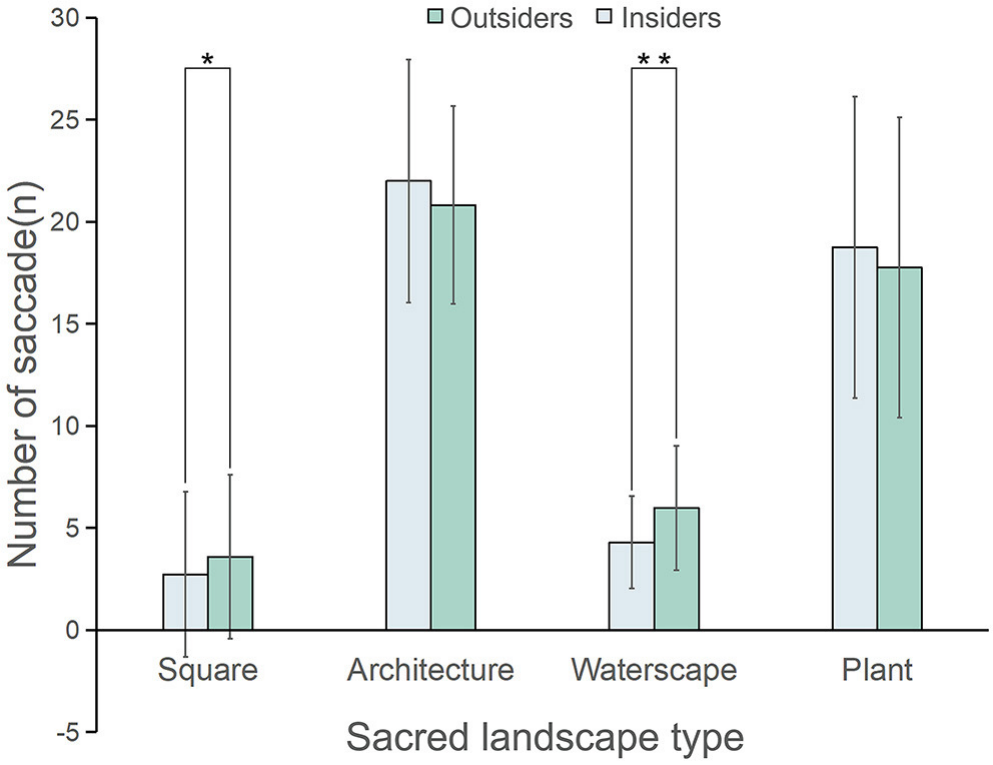
Comparison of the number of saccades of the outsiders and the insiders (*p < 0.05, **p < 0.01); the data are shown as means (SD). *N* = 45 for each group.

As shown in Table 1, the difference in the number of saccades (for all images) of the outsiders (11.94 ± 10.01) and the insiders (12.03 ± 8.93) was not significant (*z* = 0.78, *p* = 0.436, η^2^ = 0.01). Nevertheless, as presented in Figure 6, the number of saccades of the former was significantly higher in the AOI of squares (outsiders: 2.07 ± 4.05; insiders: 3.58 ± 4.01, *z* = 2.28, *p* < 0.05, η^2^ = 0.43) and waterscapes (outsiders: 4.29 ± 2.26; insiders 5.97 ± 3.05, *z* = 2.75, *p* < 0.01, η^2^ = 0.63), however, architectures and plants were excluded.

### Landscape Preference Scores of the Photos and the Corresponding Eye Movements

The correlations between the sacred landscape preference scores and the eye movement metrics (for all images) were calculated (see Table 2). We found significant positive correlations between the landscape preference scores and three eye movement metrics (the time to first fixation, the average fixation duration, and the number of saccades); however, the outsiders' number of saccades was excluded. This indicated that a higher preference score of the photos was associated with a longer time to first fixation (outsiders: *r* = 0.329, *p* < 0.01; insiders: *r* = *0.569, p* < 0.01), lengthier average fixation duration (outsiders: *r* = 0.350, *p* < 0.05; insiders: *r* = 0.539, *p* < 0.01), and more saccades (insiders: *r* = 0.351, *p* < 0.05); the outsiders had lower correlations than the insiders.

**Table 2:** Correlations between the scores of landscape preferences and the time to first fixation, the average fixation duration, and the number of saccades.

	**Preference score**	
	**Outsiders**	**Insiders**
Time to first fixation (ms)	0.392**	0.569**
Average fixation duration (ms)	0.350*	0.539**
Number of saccade (n)	0.137	0.351*

*N = 45 for each group.*

* *p < 0.05;*

** *p < 0.01*.

### Frequency of the Reasons for the Landscape Preference Scores

The researcher summarized the reasons of the outsiders and the insiders for their preference of sacred landscapes (see Table 3). Overall, 23 categories for scoring the reasons were developed, and the frequency in the sample was above 10% (*N* > 5). Furthermore, nine of these 23 categories represented the consensus for the preferences of the outsiders and the insiders for the sacred landscapes, such as relaxation (outsiders: *N* = 12; insiders: *N* = 24), spaciousness (outsiders: *N* = 10; insiders: *N* = 15), traditional (outsiders: *N* = 16; insiders: *N* = 18), and quiet (outsiders: *N* = 14; insiders: *N* = 6).

**Table 3:** Participants' reactions to the sacred landscape.

**Category**	**Common impression**		**Unique impression**	
	**Outsiders**	**Insiders**	**Outsiders**	**Insiders**
Total	Relaxation (12)	Relaxation (24)	Spirituality (12)	Sacred (21)
	Order (12)	Order (10)		Sense of security (5)
	Beautiful (9)	Beautiful (4)		
Square	Spaciousness (10)	Spaciousness (15)	Ornament (7)	Maintenance (14) Rest facilities (8)
Architecture	Traditional (16)	Traditional (18)	Construction (13)	Sense of ritual (12)
	Color (12)	Color (8)	Material (8)	Local style (16)
	Solemn (11)	Solemn (6)		
Waterscape	Quiet (14)	Quiet (6)	Vitality (7)	Watching fish (15)
				Rockery (5)
Plant	Green (12)	Green (6)	Ecology (18)	Ancient tree (18)

*N = 45 for each group*.

The participants considered religious architectures as visual landmarks that distinguished sacred landscapes from urban parks. Based on the interviews, both outsiders and insiders appreciated religious architectures in fast-changing cities. Additionally, the traditional forms of religious architectures that were specifically mentioned are as follows:

*The building in this photo has a unique regional style that is relatively different from those in the park. It gives me a sense of peace and is incredibly interesting. (Outsider ID. 21)*

*This building has a wooden structure and yellow walls, which is similar to the temples I visited as a child. I like architectures that maintain a traditional style and have not been changed by the development of the modern society. (Insider ID. 15)*

In addition, six categories represented the unique reasons for the outsiders' preference for sacred landscapes, such as spirituality (*N* = 12), construction (*N* = 13), and ecology (*N* = 18). Further, the corresponding nine categories for the insiders included sacred (*N* = 21), maintenance (*N* = 14), local style (*N* = 16), watching fish (*N* = 15), and ancient tree (*N* = 18) among others.

## Discussion

### Frequency of the Reasons for the Landscape Preference Scores

One purpose of this research was to explore the consensus on the visual preferences of the outsiders and the insiders when viewing photos of sacred landscapes. Our results demonstrated that when they observed and evaluated the various types of these landscapes, the rankings of their preference scores and eye movement metrics were consistent. In general, the visual preference scores of the sacred landscapes in descending order were architectures, waterscapes, plants, and squares. Furthermore, these scores of the outsiders and the insiders were positively correlated with the eye movement metrics (the time to first fixation, the average fixation duration, and the number of saccades).

This research's most significant finding was that people visually preferred artificial religious architectures rather than natural landscapes, as highlighted by the eye tracking data (e.g., first fixation duration, average fixation duration) and the preference scores of outsiders and insiders. Similarly, in the interview, the participants (outsiders and insiders) agreed that the Buddhist architecture with a regional style was essential to the attractiveness of sacred landscapes beyond secular spaces (e.g., park).

This result could be attributed to several aspects. First, the Buddhist architecture in the stimulus material had a sacred environmental attractiveness due to the large-scale physical structure. Previous studies found that massive physical structures were capable of eliciting feelings of awe, thereby triggering emotions related to spirituality and sacredness (Herzog et al., 2013, Ysseldyk et al., 2016).

Secondly, as an experimental stimulus, the Buddhist architecture followed the historical style, and was influenced by regional traditions with rich colors, texture levels, and easy identification. According to the quantitative theory of aesthetics, images that were effortless to process, however, were visually complex had a higher aesthetic value (Birkhoff, 1933). In addition, previous studies also found that the average fixation time was positively correlated with aesthetic preferences (Holmes and Zanker, 2012). Noticeably, the Buddhist architecture with the longest average fixation duration among the four types of sacred landscapes also had the highest aesthetic value, which was a main reason for people's preference. The results of the study confirmed the restorative effect of historical buildings based on the fascination dimension from another perspective. Prior research reported that as compared to modern buildings, the traditional ones with a higher degree of ornamentation had a greater perceived restorative quality (Kaplan and Kaplan, 1989; Van den Berg et al., 2016; Franěk et al., 2018).

As predicted, the study found that people visually preferred natural landscapes (waterscapes and plants) than religious squares. This result confirmed the findings of previous research that as compared to the artificial environment, the fractal complexity of the natural ones was higher; thus, its visual aesthetic preference score and the restoration effect were enhanced (Purcell et al., 2001; Wang et al., 2016; Franěk et al., 2019). From the perspective of environmental preference, the natural environment had simple visual information, and therefore, a weaker visual stimulation; however, the artificial environment's visual abundance of “making sense” and “involvement” could trigger appropriate visual interaction between people and their surroundings (Kaplan and Kaplan, 1989; Meagher, 2018).

### The Difference in the Landscape Preferences of Sacred Spaces

Previous research reported that different groups used and interacted with the environment in various ways, thus affecting how they assessed the landscape (Dramstad et al., 2006). This study expanded this argument from the perspective of sacred landscape visual preference. It revealed the significant difference in the subjective preference ratings of the four types of sacred landscapes of the outsiders and the insiders; specifically, the scores of the latter were significantly higher than the former. Especially with regard to the preference for squares, the insiders and the outsiders evaluated it positively and negatively, respectively. When they discussed about the unique impression of the city's sacred landscape, the former tended to have strong religious impressions (e.g., sacred, sense of ritual, and watching fish), while the latter had general recreational impressions and perceptions (e.g., spirituality, construction, and vitality). This result could be explained by the immersion effect. There was evidence that immersion in an identity-confirmed religious environment might stimulate positive effects (Mazumdar and Mazumdar, 2004, 2009; Ysseldyk et al., 2016). Therefore, the association of the insiders' identity with the place indicated that they had greater functional interaction with the experimental stimulus. The sacred landscape not only provided the insiders with a psychological and physical space that combined religious activities and green space leisure, but also conveyed important symbols to those who were familiar with spatial clues. For example, the square was an ordinary hard space for the outsiders; however, for the insiders, it was an important place for their periodic religious ceremonies.

The analysis of the eye movement metrics also showed that the time to first fixation and the average fixation duration of the insiders was overall less than those of the outsiders. The former indicated that the insiders had a higher visual experience, and the latter reflected that they could process and encode the viewing content easily.

Previous studies had demonstrated that the experience of visual stimulation of the AOI was high, and the time to first fixation was short; the greater attention it could attract (Guo et al., 2016) and the more the eye movement experiment's participants were aware of it, the longer was the average fixation duration (Dupont et al., 2015). Therefore, as compared with the outsiders, the insiders could more easily understand and experience the landscape because familiarity with background knowledge could effectively enhance the extraction of stimulus information.

Interestingly, the comparison of the number of saccades based on the scope of visual exploration showed that although the saccades of both the groups were more consistent regarding the architectures and the plants, there were significant differences in the squares and the waterscapes. The number of saccades of the insiders were more prominent than the outsiders, indicating higher visual exploration of this type of landscape.

We believed that the results may be attributed to the differences in the environmental interactions caused by the low visual complexity of the squares and the waterscapes as well as the discrepancies in the functional preferences of the two groups. However, the specific mechanism needs to be further explored.

Another noteworthy finding was the correlation between the landscape preference scores and the eye movement metrics. As compared with the outsiders, the insiders had higher correlations among the landscape preference scores, the eye movement metrics and the number of saccades of the sacred landscapes. This result demonstrated that as compared with young outsiders, the eye movements of the older insiders when viewing the sacred landscapes were more consistent with the evaluation of landscape preferences. Previous studies reported that when exploring general landscapes (such as parks and rural landscapes), the views of young people tended to reflect the prevailing norms, and their views on experience and emotion were more stable than those of the other age groups (Ren, 2019). This showed the influence of religious background that enabled the insiders to view and evaluate the sacred landscape environment based on the common preference standards.

### Limitations

This study had certain limitations. First, its sample included only the Chinese Buddhist temples and the Buddhists in China, the results and conclusions couldn't be extended to other sacred landscapes, such as the Christian and Islamic ones; besides, the composition of the participants did not provide completely accurate information. Future research should consider comparing the visual preferences of outsiders and insiders belonging to different religions for various sacred landscapes, as well as select more samples and analyze the possible influence of demographics (e.g., age, residence, social status, etc.) on the experimental results. Second, this research used carefully selected photos as a substitute for the real environment of the sacred landscape. As compared with previous studies on on-site experiences of sacred landscapes, viewing photos through an eye tracker in the laboratory could eliminate the interference of time, weather, and human factors as well as maintain the consistency of the stimulus content; however, the lack of immersion may have affected the experimental results' significance. In addition, previous research showed that the acoustic physical environment had a significant influence on the preferences for sacred spaces, such as bells and noise (Zhang et al., 2016). Future studies could consider using virtual reality technology to reproduce the real environment of the sacred landscape in a panoramic view, and add auditory stimulation to enhance the sense of immersion and experimental validity.

## Conclusions

Although sacred landscapes have been widely distributed in many cities as public green spaces, insufficient consideration was given to the visual consensus and the differences between various groups visiting them. Thus, this research explored the visual preferences regarding the sacred landscapes of the outsiders and the insiders. Taking into account the differences in religious beliefs, age, living environment, and functional preferences of the two groups, a combination of subjective evaluation and eye tracking methods were employed to examine the visual preferences regarding the sacred landscapes. An important finding of this research was the confirmation that visually, people prefer religious buildings visually rather than the natural landscapes that they usually prefer. Furthermore, this study supports the limited evidence of the existing research, such as the relationship between sacredness and environmental attractiveness and the aesthetic value of historical buildings and environmental restoration benefits (Herzog et al., 2013; Van den Berg et al., 2016; Franěk et al., 2018).

In addition, our eye tracking experiments revealed significant differences between the outsiders and the insiders in viewing and evaluating sacred landscapes. The functional interaction between the latter and the sacred landscapes enhances their visual immersion effect that could effectively improve their subjective preference scores for the sacred landscapes. As compared to the outsiders, the insiders had a higher visual experience, easier coding of visual information, and a larger range of visual exploration that was verified based on many eye movement metrics, such as less time to first fixation and average fixation duration and more saccades in the two landscape types, squares and waterscapes.

These results are particularly meaningful for the planning and management of sacred landscapes. In many cities, the sacred landscapes are public spaces open to all citizens, regardless of whether or they are religious. Therefore, it is extremely important that these landscapes provide the public with a physically and psychologically functional environment. Furthermore, the objective measurement of the consensus and the differences in the visual preferences of outsiders and insiders regarding sacred landscapes was insufficiently considered thus far. This research provided preliminary evidence for the exploration of new sacred landscape designs as well as an objective evaluation method for public participation in the visual transformation of the existing sacred landscapes.

## Data Availability Statement

The original contributions presented in the study are included in the article/supplementary material, further inquiries can be directed to the corresponding author/s.

## Ethics Statement

The studies involving human participants were reviewed and approved by Suzhou University of Science and Technology Ethics Committee. The patients/participants provided their written informed consent to participate in this study.

## Author Contributions

CL: Conceptualization, methodology, writing, and project administration. SG: Data curation, visualization, validation, and software. RW: Investigation. All authors contributed to the article and approved the submitted version.

## Funding

This work was funded by the National Key Research and Development Program of China (Technology Research on Conservation and Transformation Planning for the Characteristic towns and villages) [grant number 2019YFD1100700], the National Natural Science Foundation of China [grant number 51778388], and Landscape Architecture Discipline Construction Project of Suzhou University of Science and Technology.

## Conflict of Interest

The authors declare that the research was conducted in the absence of any commercial or financial relationships that could be construed as a potential conflict of interest.

## Publisher's Note

All claims expressed in this article are solely those of the authors and do not necessarily represent those of their affiliated organizations, or those of the publisher, the editors and the reviewers. Any product that may be evaluated in this article, or claim that may be made by its manufacturer, is not guaranteed or endorsed by the publisher.

## References

[B1] Amati M. Ghanbari Parmehr E. McCarthy C. Sita J. (2018). How eye-catching are natural features when walking through a park? Eye-tracking responses to videos of walks. Urban For. Urban Green. 31, 67–78. 10.1016/j.ufug.2017.12.013

[B2] Bilewicz M. Klebaniuk J. (2013). Psychological consequences of religious symbols in public space: crucifix display at a public university. J. Environ. Psychol. 35, 10–17. 10.1016/j.jenvp.2013.03.001

[B3] Birkhoff G. D. (1933). Aesthetic Measure. Cambridge, MA: Harvard University Press.

[B4] Cohen J. (1988). Statistical Power Analysis for the Behavioral Sciences, 2nd Edn. Hillsdale, NJ: Lawrence Erlbaum.

[B5] Coolican H. (2009). Research Methods and Statistics in Psychology. London: Hodder and Stoughton.

[B6] Cottet M. Vaudor L. Tronchère H. Roux-Michollet D. Augendre M. Brault V. (2018). Using gaze behavior to gain insights into the impacts of naturalness on city dwellers' perceptions and valuation of a landscape. J. Environ. Psychol. 60, 9–20. 10.1016/j.jenvp.2018.09.001

[B7] Dramstad W. E. Tveit M. S. Fjellstad W. J. Fry G. L. A. (2006). Relationships between visual landscape preferences and map-based indicators of landscape structure. Landsc. Urban Plan. 78, 465–474. 10.1016/j.landurbplan.2005.12.006

[B8] Dupont L. Antrop M. Van Eetvelde V. (2014). Eye-tracking analysis in landscape perception research: influence of photograph properties and landscape characteristics. Landsc. Res. 39, 417–432. 10.1080/01426397.2013.773966

[B9] Dupont L. Antrop M. Van Eetvelde V. (2015). Does landscape related expertise influence the visual perception of landscape photographs? Implications for participatory landscape planning and management. Landsc. Urban Plan. 141, 68–77. 10.1016/j.landurbplan.2015.05.003

[B10] Eriksson N. T. Wiklund-Gustin L. (2014). Blessed alienation: the Christian Monastery as a caring and restorative environment. Qual. Health Res. 24, 172–182. 10.1177/1049732313519708 24463632

[B11] Franěk M. PetruŽálek J. Šefara D. (2019). Eye movements in viewing urban images and natural images in diverse vegetation periods. Urban For. Urban Greening 46, 126477. 10.1016/j.ufug.2019.126477

[B12] Franěk M. Šefara D. PetruŽálek J. Cabal J. Myška K. (2018). Differences in eye movements while viewing images with various levels of restorativeness. J. Environ. Psychol. 57, 10–16. 10.1016/j.jenvp.2018.05.001

[B13] Guo F. Ding Y. Liu W. Liu C. Zhang X. (2016). Can eye-tracking data be measured to assess product design?: visual attention mechanism should be considered. Int. J. Ind. Ergonomics. 53, 229–235. 10.1016/j.ergon.2015.12.001

[B14] Herzog T. R. Gray L. E. Dunville A. M. Hicks A. M. Gilson E. A. (2013). Preference and tranquility for houses of worship. Environ. Behav. 45, 504–525. 10.1177/0013916511410422

[B15] Hollingworth A. (2009). Two forms of scene memory guide visual search: memory for scene context and memory for the binding of target object to scene location. Vis. Cogn. 17, 273–291. 10.1080/13506280802193367

[B16] Holmes T. Zanker J. M. (2012). Using an oculomotor signature as an indicator of aesthetic preference. i-Perception. 3, 426–439. 10.1068/i0448aap 23145294 PMC3485839

[B17] Joye Y. Pals R. Steg L. Evans B. L. (2013). New methods for assessing the fascinating nature of nature experiences. PLoS ONE 8, e65332. 10.1371/journal.pone.0065332 23922645 PMC3724873

[B18] Kaplan R. Kaplan S. (1989). The Experience of Nature: A Psychological Perspective. New York, NY: Cambridge University Press.

[B19] Kaplan S. (1995). The restorative benefits of nature: toward an integrative framework. J. Environ. Psychol. 15, 169–182. 10.1016/0272-4944(95)90001-2

[B20] Mazumdar S. Mazumdar S. (2004). Religion and place attachment: a study of sacred places. J. Environ. Psychol.24, 385–397. 10.1016/j.jenvp.2004.08.005

[B21] Mazumdar S. Mazumdar S. (2009). Religion, immigration, and home making in diaspora: Hindu space in Southern California. J. Environ. Psychol. 29, 256–266. 10.1016/j.jenvp.2008.07.004

[B22] Meagher B. R. (2018). Deciphering the religious orientation of a sacred space: disparate impressions of worship settings by congregants and external observers. J. Environ. Psychol. 55, 70–80. 10.1016/j.jenvp.2017.12.007

[B23] Merton R. K. (1972). Insiders and outsiders: a chapter in the sociology of knowledge. Am. J. Sociol. 78, 9–47. 10.1086/225294

[B24] Nene Y. L. (2000). Trees in ancient literature: I. The banyan tree. Asian Agri-Hist. 4, 311–314.

[B25] Nordh H. Evensen K. H. Skar M. (2017). A peaceful place in the city-a qualitative study of restorative components of the cemetery. Landsc. Urban Plan. 167, 108–117. 10.1016/j.landurbplan.2017.06.004

[B26] Nordh H. Hagerhall C. M. Holmqvist K. (2013). Tracking restorative components: patterns in eye movements as a consequence of a restorative rating task. Landsc. Res. 38, 101–116. 10.1080/01426397.2012.691468

[B27] Ode Sang Å. Tveit M. S. Pihel J. Hägerhäll C. M. (2016). Identifying cues for monitoring stewardship in Swedish pasture landscapes. Land Use Policy 53, 20–26. 10.1016/j.landusepol.2015.09.020

[B28] Ouellette P. Kaplan R. Kaplan S. (2005). The monastery as a restorative environment. J. Environ. Psychol. 25, 175–188. 10.1016/j.jenvp.2005.06.00124463632

[B29] Pihel J. Ode Sang Å. Hagerhall C. Nyström M. (2015). Expert and novice group differences in eye movements when assessing biodiversity of harvested forests. For. Policy Econ. 56, 20–26. 10.1016/j.forpol.2015.04.004

[B30] Purcell T. Peron E. Berto R. (2001). Why do preferences differ between scene types? Environ. Behav. 33, 93–106. 10.1177/00139160121972882

[B31] Ren X. (2019). Consensus in factors affecting landscape preference: a case study based on a cross-cultural comparison. J Environ Manage. 252, 109622. 10.1016/j.jenvman.2019.109622 31605910

[B32] Schaaf T. Lee C. (2006). Conserving cultural and biological diversity: the role of sacred natural sites and cultural landscapes, in UNESCO-IUCN International Conference (Paris: UNESCO).

[B33] Scott A. (2002). Assessing public perception of landscape: the LANDMAP experience. Landsc. Res. 27, 271–295. 10.1080/01426390220149520

[B34] Sevenant M. Antrop M. (2010). The use of latent classes to identify individual differences in the importance of landscape dimensions for aesthetic preference. Land Use Policy 27, 827–842. 10.1016/j.landusepol.2009.11.002

[B35] Šikl R. Svatonová H. Děchtěrenko F. Urbánek T. (2019). Visual recognition memory for scenes in aerial photographs: exploring the role of expertise. Acta Psychol. 197, 23–31. 10.1016/j.actpsy.2019.04.019 31077995

[B36] Sternberg E. M. (2009). Healing Spaces: The Science of Place and Well-being. New York, NY: Belknap Press.

[B37] Stevenson M. P. Dewhurst R. Schilhab T. Bentsen P. (2019). Cognitive restoration in children following exposure to nature: evidence from the attention network task and mobile eye tracking. Front. Psychol. 10, 10. 10.3389/fpsyg.2019.00042 30804825 PMC6370667

[B38] Tenngart Ivarsson C. Hagerhall C. M. (2008). The perceived restorativeness of gardens – assessing the restorativeness of a mixed built and natural scene type. Urban For. Urban Green. 7, 107–118. 10.1016/j.ufug.2008.01.001

[B39] Terzidou M. Scarles C. Saunders M. N. K. (2018). The complexities of religious tourism motivations: sacred places, vows and visions. Ann. Tour. Res. 70, 54–65. 10.1016/j.annals.2018.02.011

[B40] Ulrich R. S. (1979). Visual landscapes and psychological well-being. Landsc. Res. 4, 17–23. 10.1080/01426397908705892

[B41] Ulrich R. S. Simons R. F. Losito B. D. Fiorito E. Miles M. A. Zelson M. (1991). Stress recovery during exposure to natural and urban environments. J. Environ. Psychol. 11, 201–230. 10.1016/S0272-4944(05)80184-7

[B42] Van den Berg A. E. Joye Y. Koole S. L. (2016). Why viewing nature is more fascinating and restorative than viewing buildings: a closer look at perceived complexity. Urban For. Urban Green. 20, 397–401. 10.1016/j.ufug.2016.10.011

[B43] van den Berg A. E. Vlek C. A. J. Coeterier J. F. (1998). Group differences in the aesthetic evaluation of nature development plans: a multilevel approach. J. Environ. Psychol. 18, 141–157. 10.1006/jevp.1998.0080

[B44] Verschuuren B. Wild R. McNeely J. A. Oviedo G. (2010). Sacred Natural Sites: Conserving Nature and Culture. London; Washington, DC: Earthscan.

[B45] Wang X. X. Rodiek S. Wu C. Z. Chen Y. Li Y. X. (2016). Stress recovery and restorative effects of viewing different urban park scenes in Shanghai, China. Urban For. Urban Green. 15, 112–122. 10.1016/j.ufug.2015.12.003

[B46] Ysseldyk R. Haslam S. A. Morton T. A. (2016). Stairway to heaven? (Ir)religious identity moderates the effects of immersion in religious spaces on self-esteem and self-perceived physical health. J. Environ. Psychol. 47, 14–21. 10.1016/j.jenvp.2016.04.016

[B47] Zhang D. Zhang M. Liu D. Kang J. (2016). Soundscape evaluation in han chinese buddhist temples. Appl. Acoust. 111, 188–197. 10.1016/j.apacoust.2016.04.020

